# Crystal structure of 1-(4-fluoro­phen­yl)-4-(4-meth­oxy­phen­yl)-1*H*-1,2,3-triazole

**DOI:** 10.1107/S2056989015012153

**Published:** 2015-07-04

**Authors:** Balbir Kumar, Madhvi Bhardwaj, Satya Paul, Rajni Kant, Vivek K. Gupta

**Affiliations:** a Post-Graduate Department of Physics & Electronics, University of Jammu, Jammu Tawi 180 006, India; b Department of Chemistry, University of Jammu, Jammu Tawi 180 006, India

**Keywords:** crystal structure, 1,2,3-triazole, π–π inter­actions

## Abstract

In the title compound, C_15_H_12_FN_3_O, the triazole ring forms dihedral angles of 30.57 (8) and 21.81 (9)° with the fluoro-substituted and meth­oxy-substituted benzene rings, respectively. The dihedral angle between the benzene rings is 51.53 (7)°. In the crystal, π–π inter­actions between the triazole rings [centroid–centroid seperations = 3.774 (2) and 3.841 (2) Å] form chains along [010].

## Related literature   

For related literature on 1,2,3-triazoles, see: Aher *et al.* (2009 ▸); Jordao *et al.* (2009 ▸); Vijaya Raghava Reddy *et al.* (2010 ▸); Soltis *et al.* (1996 ▸). For applications of 1,2,3-triazoles, see: Pérez-Balderas *et al.* (2003 ▸); Wu *et al.* (2004 ▸); Kumar & Pandey (2008 ▸); Haridas *et al.* (2008 ▸); Turner *et al.*, (2007 ▸); Angell & Burgess (2007 ▸); For the synthesis of 1,2,3-triazoles, see: Huisgen *et al.* (1965 ▸); Wang *et al.* (2010 ▸). For related structures, see: Abdel-Wahab *et al.* (2012 ▸); Zhang *et al.* (2004 ▸).
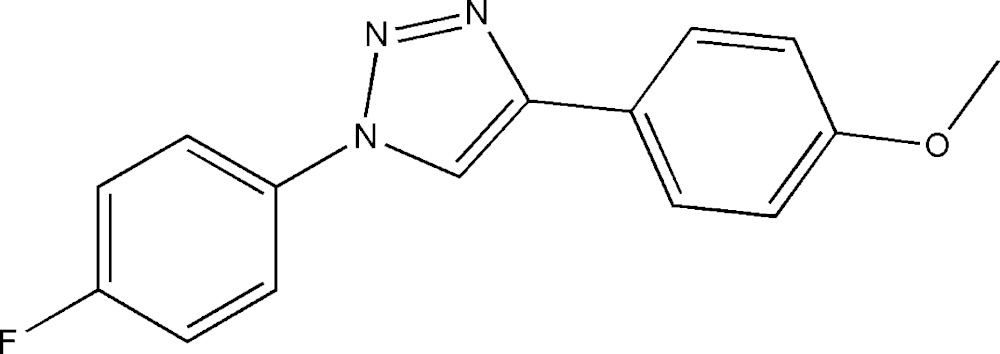


## Experimental   

### Crystal data  

C_15_H_12_FN_3_O
*M* *_r_* = 269.28
Triclinic, 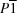

*a* = 5.6572 (5) Å
*b* = 7.3692 (8) Å
*c* = 15.5711 (15) Å
α = 79.202 (9)°
β = 81.159 (8)°
γ = 89.442 (8)°
*V* = 629.95 (11) Å^3^
*Z* = 2
Mo *K*α radiation
μ = 0.10 mm^−1^
*T* = 293 K
0.30 × 0.20 × 0.20 mm

### Data collection  

Oxford Diffraction Xcalibur Sapphire3 diffractometer
Absorption correction: multi-scan (*CrysAlis PRO*; Oxford Diffraction, 2010 ▸) *T*_min_ = 0.806, *T*_max_ = 1.000
4369 measured reflections
2461 independent reflections
1575 reflections with *I* > 2σ(*I*)
*R*_int_ = 0.034

### Refinement  

*R*[*F* ^2^ > 2σ(*F* ^2^)] = 0.057
*wR*(*F* ^2^) = 0.179
*S* = 1.04
2461 reflections
182 parameters
H-atom parameters constrained
Δρ_max_ = 0.26 e Å^−3^
Δρ_min_ = −0.22 e Å^−3^

### 

Data collection: *CrysAlis PRO* (Oxford Diffraction, 2010 ▸); cell refinement: *CrysAlis PRO*; data reduction: *CrysAlis PRO*; program(s) used to solve structure: *SHELXS97* (Sheldrick, 2008 ▸); program(s) used to refine structure: *SHELXL97* (Sheldrick, 2008 ▸); molecular graphics: *ORTEP-3 for Windows* (Farrugia, 2012 ▸); software used to prepare material for publication: *PLATON* (Spek, 2009 ▸).

## Supplementary Material

Crystal structure: contains datablock(s) I, New_Global_Publ_Block. DOI: 10.1107/S2056989015012153/lh5772sup1.cif

Structure factors: contains datablock(s) I. DOI: 10.1107/S2056989015012153/lh5772Isup2.hkl

Supporting information file. DOI: 10.1107/S2056989015012153/lh5772Isup3.cml

**Fig. 1.**
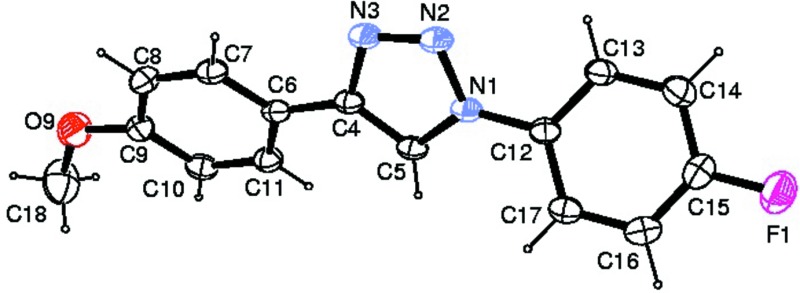
The molecular structure of the title compound with displacement ellipsoids drawn at the 40% probability level. H atoms are shown as small spheres of arbitrary radii.

**Fig. 2.**
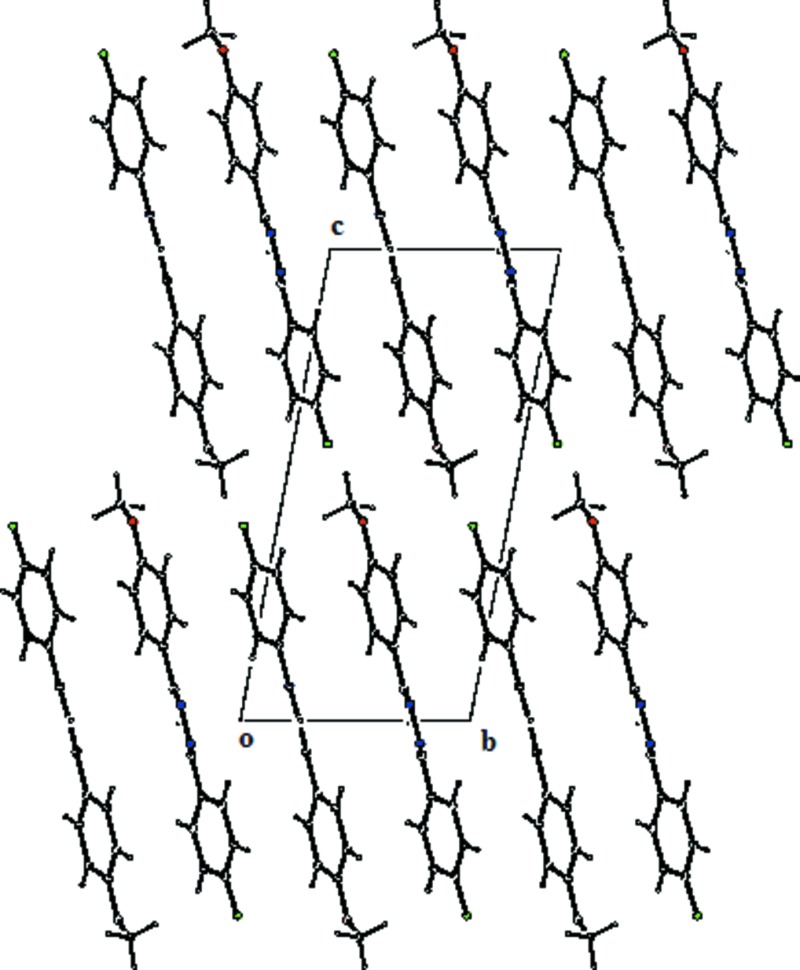
The packing arrangement of molecules viewed along the a axis.

. DOI: 10.1107/S2056989015012153/lh5772fig1.tif
The mol­ecular structure of the title compound with displacement ellipsoids drawn at the 40% probability level. H atoms are shown as small spheres of arbitrary radii.

a . DOI: 10.1107/S2056989015012153/lh5772fig2.tif
The packing arrangement of mol­ecules viewed along the *a* axis.

CCDC reference: 1408544

Additional supporting information:  crystallographic information; 3D view; checkCIF report

## supplementary crystallographic information

### S1. Comment

1,2,3-Triazoles are an important class of organic compounds which have become prominent in recent years as superbly versatile five membered nitrogen heterocycles. The 1,2,3-triazole family exhibit a broad spectrum of bioactivities such as antifungal (Aher *et al.*, 2009) antiviral (Jordao *et al.*, 2009), antibacterial (Vijaya Raghava Reddy *et al.*, 2010) and anticancer (Soltis *et al.*, 1996) activities. Furthermore 1,4-disubstituted 1,2,3-triazoles have also been used as a ligation tool for the synthesis of neoglyco-conjugates (Perez-Balderas *et al.*, 2003), multivalent dendrimeric peptides (Wu *et al.*, 2004), ionic receptors (Kumar *et al.*, 2008), triazolophanes (Haridas *et al.*, 2008), cyclic peptides (Turner *et al.*, 2007) and peptidomimetics (Angell *et al.*, 2007). 1,2,3-Triazoles are traditionally obtained using the thermal 1,3-dipolar cycloaddition of organic azides with alkynes (Huisgen *et al.*, 1965) that has been known for nearly five decades. Recently, copper based catalysis was found to dramatically accelerate the reaction under mild conditions while achieving a high regioselectivity towards the 1,4-regioisomer of the triazole product (Wang *et al.*, 2010). This powerful, highly reliable, and selective reaction is the paradigm of a click reaction, which placed it in a class of its own and has enabled many novel applications.

The molecular structure of the title compound is shown in Fig. 1. The triazole ring forms dihedral angles of 30.57 (8)° and 21.81 (9)° with the fluoro-substituted and methoxy-substituted benzen rings, respectively. The dihedral angle between the benzene rings is 51.53 (7)°. All bond lengths and angles are normal and correspond to those observed in the related structures (Zhang *et al.*, 2004; Abdel-Wahab *et al.*, 2012). The C15—F1 bond length [1.357 (4) Å] agrees well with the accepted value of 1.340 Å for the F-C_aromatic_ length and is in good agreement with a structure of this type (Abdel-Wahab *et al.*, 2012). In the crystal, π–π interactions observed between the triazole rings [centroid–centroid seperations = 3.774 (2) and 3.841 (2) Å] form chains along [010] (Fig. 2).

### S2. Experimental

Synthesis of 1-(4-flourophenyl)-4-(4-methoxyphenyl) -1*H*-1,2,3-triazole: To 4-fluoroaniline (0.22 g, 2 mmol) in a round bottomed flask maintained at 273-278 K, mixture of conc. HCl: H_2_O (1.5 ml, 1:1) was added and stirred for 5 min. Then solution of NaNO_2_ (0.17 g, 2.5 mmol in 1 ml water) was added dropwise over a period of 5 min. After stirring for another 5 min, sodium azide (0.19 g, 3 mmol) was added and the reaction mixture was further stirred for another 10 min. Finally, 4-methoxyphenylacetylene (0.19 g, 1.5 mmol) and catalyst [Cu(0)-Fe_3_O_4_@SiO_2_/NH_2_Cel] (0.05 g) were added to the reaction mixture followed by stirring at room temperature for 6 h. The reaction was then stopped and the catalyst was separated using an external magnet. The reaction mixture was extracted with EtOAc, washed with water and dried over Na_2_SO_4_. Finally, the product was obtained after removal of the solvent under reduced pressure followed by crystallization with EtOAc: pet ether. The product, 1-(4-flourophenyl)-4-(4-methoxyphenyl) -1*H*-1,2,3-triazole was obtained as shiny white crystals.

### S3. Refinement

All H atoms were geometrically fixed and allowed to ride on their parent C atoms, with C—H distances of 0.93–0.96 Å; and with *U*_iso_(H) = 1.2*U*_eq_(C), except for the methyl group where *U*_iso_(H) = 1.5*U*_eq_(C).

### Crystal data

**Table:**

C_15_H_12_FN_3_O	*Z* = 2
*M**_r_* = 269.28	*F*(000) = 280
Triclinic, *P*1	*D*_x_ = 1.420 Mg m^−^^3^*D*_m_ = 1.42 Mg m^−^^3^*D*_m_ measured by not measured
Hall symbol: -P 1	Mo *K*α radiation, λ = 0.71073 Å
*a* = 5.6572 (5) Å	Cell parameters from 1205 reflections
*b* = 7.3692 (8) Å	θ = 4.0–28.0°
*c* = 15.5711 (15) Å	µ = 0.10 mm^−^^1^
α = 79.202 (9)°	*T* = 293 K
β = 81.159 (8)°	Block, white
γ = 89.442 (8)°	0.30 × 0.20 × 0.20 mm
*V* = 629.95 (11) Å^3^	

### Data collection

**Table:**

Oxford Diffraction Xcalibur Sapphire3 diffractometer	2461 independent reflections
Radiation source: fine-focus sealed tube	1575 reflections with *I* > 2σ(*I*)
Graphite monochromator	*R*_int_ = 0.034
Detector resolution: 16.1049 pixels mm^-1^	θ_max_ = 26.0°, θ_min_ = 3.7°
ω scans	*h* = −4→6
Absorption correction: multi-scan (*CrysAlis PRO*; Oxford Diffraction, 2010)	*k* = −7→9
*T*_min_ = 0.806, *T*_max_ = 1.000	*l* = −18→19
4369 measured reflections	

### Refinement

**Table:**

Refinement on *F*^2^	Primary atom site location: structure-invariant direct methods
Least-squares matrix: full	Secondary atom site location: difference Fourier map
*R*[*F*^2^ > 2σ(*F*^2^)] = 0.057	Hydrogen site location: inferred from neighbouring sites
*wR*(*F*^2^) = 0.179	H-atom parameters constrained
*S* = 1.04	*w* = 1/[σ^2^(*F*_o_^2^) + (0.0744*P*)^2^ + 0.0652*P*] where *P* = (*F*_o_^2^ + 2*F*_c_^2^)/3
2461 reflections	(Δ/σ)_max_ < 0.001
182 parameters	Δρ_max_ = 0.26 e Å^−^^3^
0 restraints	Δρ_min_ = −0.22 e Å^−^^3^

### Special details

**Table:**

Geometry. All e.s.d.'s (except the e.s.d. in the dihedral angle between two l.s. planes) are estimated using the full covariance matrix. The cell e.s.d.'s are taken into account individually in the estimation of e.s.d.'s in distances, angles and torsion angles; correlations between e.s.d.'s in cell parameters are only used when they are defined by crystal symmetry. An approximate (isotropic) treatment of cell e.s.d.'s is used for estimating e.s.d.'s involving l.s. planes.
Refinement. Refinement of *F*^2^ against ALL reflections. The weighted *R*-factor *wR* and goodness of fit *S* are based on *F*^2^, conventional *R*-factors *R* are based on *F*, with *F* set to zero for negative *F*^2^. The threshold expression of *F*^2^ > σ(*F*^2^) is used only for calculating *R*-factors(gt) *etc*. and is not relevant to the choice of reflections for refinement. *R*-factors based on *F*^2^ are statistically about twice as large as those based on *F*, and *R*- factors based on ALL data will be even larger.

### Fractional atomic coordinates and isotropic or equivalent isotropic displacement parameters (Å^2^)

**Table:**

	*x*	*y*	*z*	*U*_iso_*/*U*_eq_	
F1	0.2052 (4)	−0.1487 (2)	0.41281 (12)	0.0782 (6)	
N1	0.1232 (4)	0.1852 (3)	0.07275 (13)	0.0404 (5)	
N2	−0.0970 (4)	0.1982 (3)	0.04842 (15)	0.0505 (6)	
N3	−0.0714 (4)	0.2743 (3)	−0.03467 (15)	0.0489 (6)	
C4	0.1657 (4)	0.3132 (3)	−0.06593 (16)	0.0367 (6)	
C5	0.2893 (4)	0.2552 (3)	0.00235 (15)	0.0395 (6)	
H5	0.4540	0.2623	0.0009	0.047*	
C6	0.2436 (4)	0.3971 (3)	−0.15737 (16)	0.0371 (6)	
C7	0.1015 (5)	0.3849 (3)	−0.22101 (17)	0.0426 (6)	
H7	−0.0454	0.3228	−0.2040	0.051*	
C8	0.1707 (5)	0.4613 (4)	−0.30769 (18)	0.0483 (7)	
H8	0.0717	0.4501	−0.3490	0.058*	
C9	0.3880 (5)	0.5558 (3)	−0.33479 (17)	0.0457 (7)	
O9	0.4360 (4)	0.6328 (3)	−0.42195 (14)	0.0718 (7)	
C10	0.5341 (5)	0.5698 (3)	−0.27319 (17)	0.0452 (6)	
H10	0.6804	0.6327	−0.2906	0.054*	
C11	0.4626 (5)	0.4900 (3)	−0.18530 (16)	0.0414 (6)	
H11	0.5629	0.4987	−0.1441	0.050*	
C12	0.1484 (4)	0.1035 (3)	0.16056 (16)	0.0371 (6)	
C13	−0.0340 (5)	0.1177 (3)	0.22842 (16)	0.0430 (6)	
H13	−0.1694	0.1844	0.2166	0.052*	
C14	−0.0162 (5)	0.0338 (4)	0.31322 (18)	0.0518 (7)	
H14	−0.1391	0.0417	0.3594	0.062*	
C15	0.1878 (5)	−0.0630 (3)	0.32897 (18)	0.0500 (7)	
C16	0.3719 (5)	−0.0751 (3)	0.26282 (19)	0.0503 (7)	
H16	0.5088	−0.1392	0.2752	0.060*	
C17	0.3531 (5)	0.0081 (3)	0.17797 (17)	0.0425 (6)	
H17	0.4773	0.0007	0.1322	0.051*	
C18	0.6617 (8)	0.6997 (5)	−0.4596 (2)	0.0918 (12)	
H18A	0.7031	0.7988	−0.4321	0.138*	
H18B	0.6648	0.7446	−0.5218	0.138*	
H18C	0.7749	0.6025	−0.4512	0.138*	

### Atomic displacement parameters (Å^2^)

**Table:**

	*U*^11^	*U*^22^	*U*^33^	*U*^12^	*U*^13^	*U*^23^
F1	0.0830 (15)	0.0859 (12)	0.0607 (12)	0.0042 (11)	−0.0198 (10)	0.0062 (9)
N1	0.0268 (11)	0.0459 (12)	0.0481 (13)	0.0005 (9)	−0.0039 (9)	−0.0096 (9)
N2	0.0252 (11)	0.0691 (14)	0.0558 (15)	−0.0006 (10)	−0.0048 (10)	−0.0098 (11)
N3	0.0260 (12)	0.0675 (15)	0.0508 (14)	−0.0006 (11)	−0.0033 (10)	−0.0069 (11)
C4	0.0268 (13)	0.0389 (12)	0.0455 (15)	0.0003 (10)	−0.0056 (10)	−0.0105 (10)
C5	0.0224 (12)	0.0470 (14)	0.0483 (15)	−0.0018 (11)	−0.0025 (10)	−0.0090 (11)
C6	0.0279 (13)	0.0391 (12)	0.0458 (15)	0.0054 (10)	−0.0075 (11)	−0.0108 (10)
C7	0.0303 (13)	0.0450 (13)	0.0536 (16)	−0.0001 (11)	−0.0086 (11)	−0.0107 (11)
C8	0.0393 (15)	0.0583 (16)	0.0513 (17)	0.0049 (13)	−0.0158 (13)	−0.0136 (12)
C9	0.0450 (16)	0.0499 (14)	0.0404 (15)	0.0101 (13)	−0.0063 (12)	−0.0049 (11)
O9	0.0608 (14)	0.0911 (15)	0.0542 (13)	−0.0043 (12)	−0.0021 (11)	0.0045 (11)
C10	0.0321 (14)	0.0458 (14)	0.0553 (17)	−0.0029 (12)	0.0001 (12)	−0.0083 (12)
C11	0.0331 (14)	0.0447 (13)	0.0484 (15)	0.0003 (11)	−0.0088 (11)	−0.0120 (11)
C12	0.0293 (13)	0.0358 (12)	0.0458 (15)	−0.0034 (10)	−0.0048 (11)	−0.0070 (10)
C13	0.0316 (13)	0.0452 (14)	0.0497 (16)	0.0032 (11)	−0.0020 (11)	−0.0063 (11)
C14	0.0441 (16)	0.0556 (16)	0.0522 (18)	0.0000 (14)	0.0027 (13)	−0.0092 (12)
C15	0.0532 (18)	0.0471 (15)	0.0493 (17)	−0.0049 (13)	−0.0165 (14)	−0.0011 (12)
C16	0.0401 (15)	0.0457 (14)	0.0657 (19)	0.0059 (12)	−0.0131 (14)	−0.0079 (13)
C17	0.0317 (13)	0.0425 (13)	0.0535 (16)	0.0001 (11)	−0.0038 (11)	−0.0121 (11)
C18	0.091 (3)	0.109 (3)	0.068 (2)	−0.032 (2)	0.000 (2)	−0.0060 (19)

### Geometric parameters (Å, º)

**Table:**

F1—C15	1.357 (3)	O9—C18	1.376 (4)
N1—C5	1.354 (3)	C10—C11	1.384 (3)
N1—N2	1.354 (3)	C10—H10	0.9300
N1—C12	1.415 (3)	C11—H11	0.9300
N2—N3	1.297 (3)	C12—C13	1.377 (3)
N3—C4	1.368 (3)	C12—C17	1.385 (3)
C4—C5	1.362 (3)	C13—C14	1.367 (3)
C4—C6	1.444 (3)	C13—H13	0.9300
C5—H5	0.9300	C14—C15	1.381 (4)
C6—C7	1.384 (3)	C14—H14	0.9300
C6—C11	1.390 (3)	C15—C16	1.363 (4)
C7—C8	1.360 (3)	C16—C17	1.368 (3)
C7—H7	0.9300	C16—H16	0.9300
C8—C9	1.387 (4)	C17—H17	0.9300
C8—H8	0.9300	C18—H18A	0.9600
C9—O9	1.356 (3)	C18—H18B	0.9600
C9—C10	1.377 (4)	C18—H18C	0.9600
			
C5—N1—N2	109.5 (2)	C10—C11—C6	121.3 (2)
C5—N1—C12	130.8 (2)	C10—C11—H11	119.4
N2—N1—C12	119.7 (2)	C6—C11—H11	119.4
N3—N2—N1	107.7 (2)	C13—C12—C17	120.3 (2)
N2—N3—C4	109.6 (2)	C13—C12—N1	119.3 (2)
C5—C4—N3	107.4 (2)	C17—C12—N1	120.4 (2)
C5—C4—C6	131.8 (2)	C14—C13—C12	120.1 (2)
N3—C4—C6	120.8 (2)	C14—C13—H13	120.0
N1—C5—C4	105.8 (2)	C12—C13—H13	120.0
N1—C5—H5	127.1	C13—C14—C15	118.7 (2)
C4—C5—H5	127.1	C13—C14—H14	120.7
C7—C6—C11	117.5 (2)	C15—C14—H14	120.7
C7—C6—C4	120.5 (2)	F1—C15—C16	119.1 (2)
C11—C6—C4	122.0 (2)	F1—C15—C14	119.0 (3)
C8—C7—C6	121.9 (3)	C16—C15—C14	121.9 (3)
C8—C7—H7	119.1	C15—C16—C17	119.2 (2)
C6—C7—H7	119.1	C15—C16—H16	120.4
C7—C8—C9	120.3 (2)	C17—C16—H16	120.4
C7—C8—H8	119.9	C16—C17—C12	119.7 (2)
C9—C8—H8	119.9	C16—C17—H17	120.1
O9—C9—C10	125.0 (3)	C12—C17—H17	120.1
O9—C9—C8	115.6 (3)	O9—C18—H18A	109.5
C10—C9—C8	119.3 (2)	O9—C18—H18B	109.5
C9—O9—C18	120.7 (3)	H18A—C18—H18B	109.5
C9—C10—C11	119.8 (3)	O9—C18—H18C	109.5
C9—C10—H10	120.1	H18A—C18—H18C	109.5
C11—C10—H10	120.1	H18B—C18—H18C	109.5
			
C5—N1—N2—N3	−0.1 (3)	O9—C9—C10—C11	178.0 (2)
C12—N1—N2—N3	−178.86 (19)	C8—C9—C10—C11	−0.1 (4)
N1—N2—N3—C4	−0.3 (3)	C9—C10—C11—C6	−0.7 (4)
N2—N3—C4—C5	0.6 (3)	C7—C6—C11—C10	1.0 (3)
N2—N3—C4—C6	179.3 (2)	C4—C6—C11—C10	−179.9 (2)
N2—N1—C5—C4	0.5 (2)	C5—N1—C12—C13	150.9 (2)
C12—N1—C5—C4	179.1 (2)	N2—N1—C12—C13	−30.7 (3)
N3—C4—C5—N1	−0.7 (2)	C5—N1—C12—C17	−30.0 (3)
C6—C4—C5—N1	−179.1 (2)	N2—N1—C12—C17	148.5 (2)
C5—C4—C6—C7	156.9 (2)	C17—C12—C13—C14	−1.6 (4)
N3—C4—C6—C7	−21.4 (3)	N1—C12—C13—C14	177.6 (2)
C5—C4—C6—C11	−22.2 (4)	C12—C13—C14—C15	0.6 (4)
N3—C4—C6—C11	159.5 (2)	C13—C14—C15—F1	−179.1 (2)
C11—C6—C7—C8	−0.4 (3)	C13—C14—C15—C16	0.8 (4)
C4—C6—C7—C8	−179.5 (2)	F1—C15—C16—C17	178.8 (2)
C6—C7—C8—C9	−0.4 (4)	C14—C15—C16—C17	−1.2 (4)
C7—C8—C9—O9	−177.6 (2)	C15—C16—C17—C12	0.1 (4)
C7—C8—C9—C10	0.7 (4)	C13—C12—C17—C16	1.2 (4)
C10—C9—O9—C18	14.0 (4)	N1—C12—C17—C16	−177.9 (2)
C8—C9—O9—C18	−167.9 (3)		
