# Supplementary figures and images for: Crystal structure of 1-(4-fluoro­phen­yl)-4-(4-meth­oxy­phen­yl)-1H-1,2,3-triazole
Source: Acta Crystallogr E Crystallogr Commun. 2015 Jul 4;71(Pt 8):o534–5. doi: 10.1107/S2056989015012153 (PMC4571383; doi:10.1107/S2056989015012153)

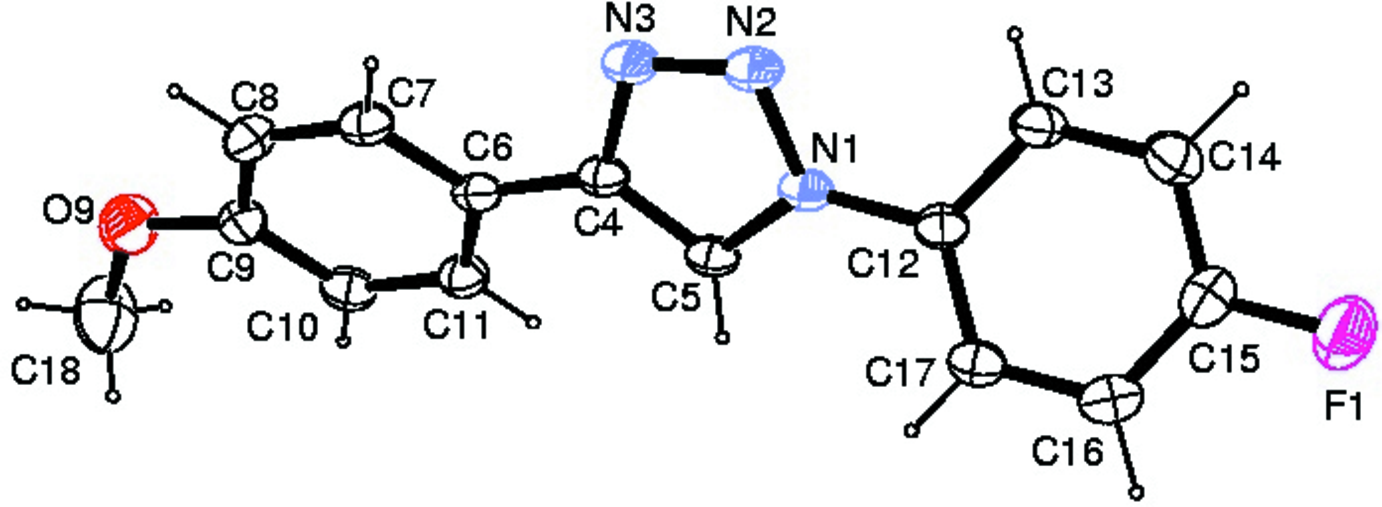

Supplement: Supplementary file 4 [file e-71-0o534-fig1.tif]

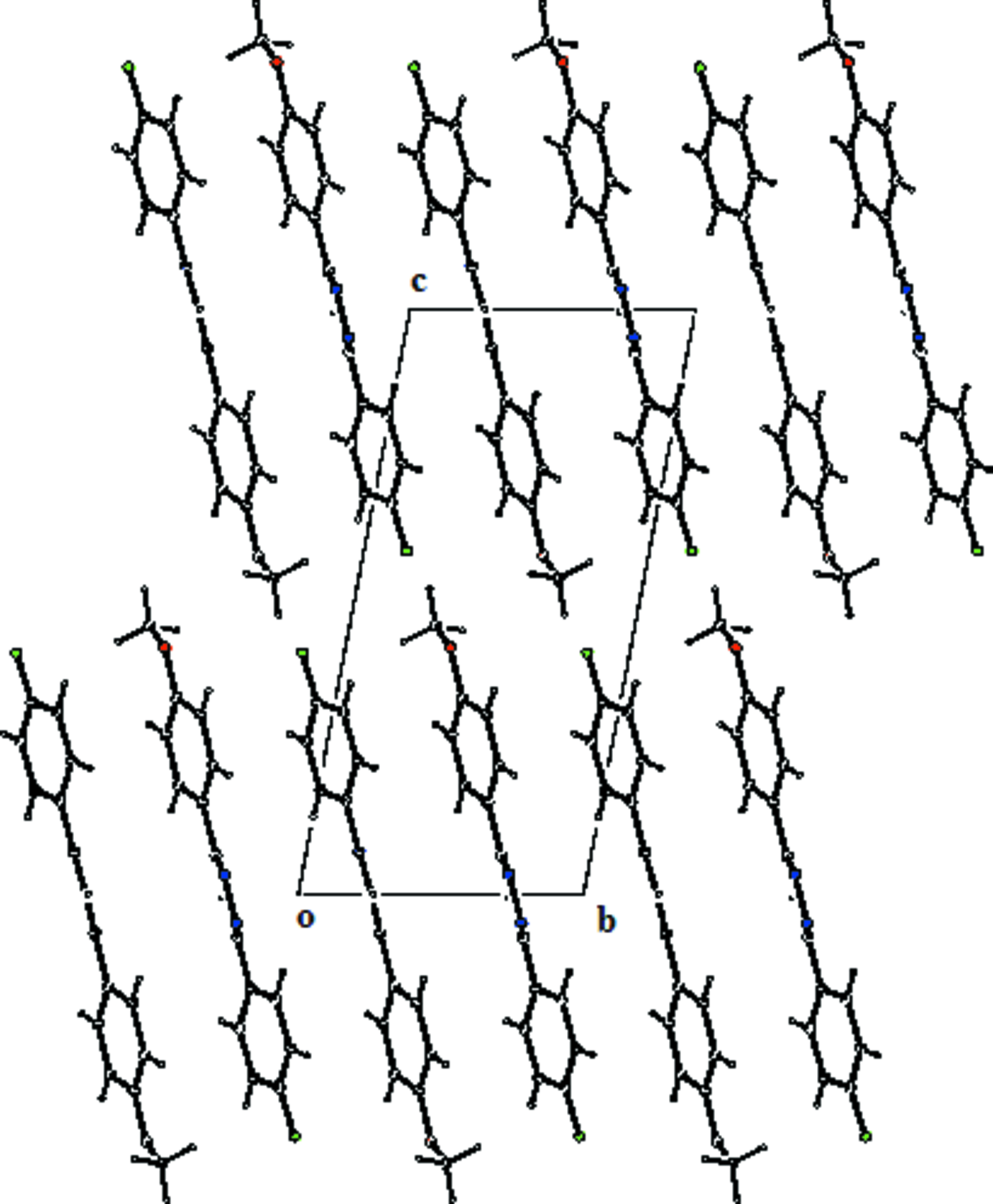

Supplement: Supplementary file 5 [file e-71-0o534-fig2.tif]
